# Four-dimensional flow provides incremental diagnostic value over echocardiography in aortic stenosis

**DOI:** 10.1136/openhrt-2024-003081

**Published:** 2025-05-07

**Authors:** Ciaran Grafton-Clarke, Hosamadin Assadi, Rui Li, Zia Mehmood, Rimma Hall, Gareth Matthews, Vasiliki Tsampasian, Samer Alabed, Bahman Kasmai, Laura Staff, John Curtin, Gurung-Koney Yashoda, Julia Sun, Sunil Nair, David Hewson, Kurian Thampi, Jordi Broncano, Fabrizio Ricci, Peter Swoboda, Andrew J Swift, Vassilios S Vassiliou, Rob J van der Geest, Pankaj Garg

**Affiliations:** 1 Norfolk and Norwich University Hospitals NHS Foundation Trust, Norwich, UK; 2 Department of Cardiovascular and Metabolic Health, University of East Anglia, Norwich, UK; 3 Department of Infection, Immunity and Cardiovascular Disease, University of Sheffield, Sheffield, UK; 4 Hospital San Juan de Dios, Santa Cruz de Tenerife, Spain; 5 Department of Neuroscience, Imaging and Clinical Sciences, Chieti and University Cardiology Division, Chieti, Italy; 6 LICAMM, University of Leeds, Leeds, UK; 7 Department of Radiology, Division of Image Processing, Leiden University Medical Center (LUMC), Leiden, The Netherlands

**Keywords:** Magnetic Resonance Imaging, Echocardiography, Aortic Valve Stenosis

## Abstract

**Aims:**

Four-dimensional flow cardiovascular MRI (4D flow CMR) has emerged as a promising technique for assessing aortic stenosis (AS). This study aimed to evaluate the agreement between 4D flow CMR and transthoracic echocardiography (TTE) in estimating peak aortic valve (AV) velocities (V_Peak_), grading AS severity and predicting AV intervention in a real-world setting.

**Methods:**

Participants from the PREFER-CMR registry who had consecutive TTE and 4D flow CMR were included. AS severity was graded using established protocols using three echocardiographic parameters (V_Peak_, AV area and mean pressure gradient) and CMR-derived V_Peak_.

**Results:**

The study recruited 30 patients (mean age 75.4 years, 67% male), with 17 undergoing AV intervention. Continuous wave Doppler (CWD) V_Peak_ (3.4 vs 2.6 m/s, p=0.0025) and 4D flow V_Peak_ (4.2 vs 2.7 m/s, p<0.0001) were significantly higher in patients going for AV intervention. V_Peak_ by CWD was significantly lower to 4D flow with a bias of −0.5 (p=0.01) and a correlation of (R=0.55, p=0.002). The Cox-regression analysis reveals that 4D flow V_Peak_ significantly predicts AV intervention (HR=2.51, p<0.01), while CWD V_Peak_ (HR=0.54, p=0.76) shows no significant association; overall model fit is significant (χ²=9.5, p=0.02).

**Conclusion:**

4D flow CMR-derived V_Peak_ assessment is superior to echocardiographic CWD assessment for predicting timing of AV intervention.

**Trial registration number:**

NCT05114785.

**WHAT IS ALREADY KNOWN ON THIS TOPIC**

- Transthoracic echocardiography (TTE) is the standard for aortic stenosis (AS) assessment but has limitations like Doppler misalignment and flow-dependence, affecting accuracy. Four-dimensional flow cardiovascular MRI (4D flow CMR) offers advanced three-dimensional blood flow visualisation, reducing alignment errors and showing potential in AS evaluation.

**WHAT THIS STUDY ADDS**

- Predictive accuracy: 4D flow CMR peak aortic valve (AV) velocity (V_Peak_) outperforms TTE in predicting AV intervention (HR=2.51, p<0.01). Threshold insight: identifies >3.5 m/s 4D flow Vpeak as a robust marker for intervention needs.

**HOW THIS STUDY MIGHT AFFECT RESEARCH, PRACTICE OR POLICY**

- Clinical adoption: Advocates 4D flow CMR for improved AS diagnosis, especially in discordant TTE cases. Guideline evolution: supports integrating 4D flow CMR into AS management protocols for better outcomes.

## Introduction

 Aortic valve (AV) stenosis (AS) is a common health burden, affecting 5% of people aged above 75 years.^1^ Accurate assessment of AS is crucial in clinical practice, as therapeutic decision-making and prognostication depend on it.^2^

Transthoracic echocardiography (TTE) is the recommended imaging modality for diagnosing AS, assessing haemodynamic severity and evaluating the prognosis and timing of valve intervention.^3 4^ Peak velocity of blood flow across the AV, as measured using continuous wave doppler (CWD) ultrasound, is a crucial parameter for clinical decision-making in patients with AS. It is the strongest echocardiographic predictor of symptom development and adverse outcomes, with higher velocities conferring increased risk.^5 6^ For instance, peak aortic jet velocity plays a significant role in the decision to proceed with surgical or transcatheter valve replacement for asymptomatic patients with severe AS.^4 7^ Furthermore, it is an essential component of risk stratification for AS, as higher peak aortic jet velocity is associated with increased risk of adverse outcomes, including heart failure and mortality.^8^

However, the accuracy of TTE assessment can be affected by a range of patient, operator and technical factors. Misalignment of the ultrasound beam with the AS jet can lead to a significant underestimation of peak jet velocity, and the eccentricity of the jet resulting from restriction along the leaflet coaptation edges can make it challenging to align the CWD ultrasound beam parallel to the jet.^2 3^ Another limitation is that peak aortic jet velocity is highly flow-dependent, which can overestimate AS severity in high-flow states such as concurrent aortic regurgitation, severe anaemia and thyrotoxicosis.^3^ Therefore, careful attention to image quality and the impact of various technical factors is essential to ensure accurate and reliable measurement of peak AV velocity (V_Peak_) in clinical practice.

Four-dimensional flow cardiovascular MRI (4D flow CMR) has emerged as a novel imaging technique for AS assessment, allowing quantification of cross-sectional planar velocities throughout the cardiac cycle.^9^ It offers several advantages over traditional sonographic assessment. First, 4D flow CMR provides a complete spatiotemporal representation of blood flow, allowing for the quantification of cross-sectional planar velocities throughout the cardiac cycle. This reduces the risk of misalignment errors that can occur with Doppler TTE.^10^ Second, 4D flow CMR enables the identification of the location of maximum velocity in three-dimensional space, which is a significant advantage over both Doppler TTE and standard two-dimensional phase-contrast CMR.^11^ Lastly, research evidence suggests that 4D flow CMR-derived valve metrics are more closely associated with invasively-obtained estimates of pressure gradient assessment than CWD TTE.^9^ These features make 4D flow CMR a powerful tool in the comprehensive, yet accurate and reproducible assessment of AS.

Patients with AS require accurate diagnostic tools to guide therapeutic decision-making, whether this involves medical management, surgical AV replacement or transcatheter AV implantation. This study aimed to evaluate the concordance of 4D flow CMR and TTE to estimate V_Peak_ and grade AS severity. Second, this study evaluated the utility of 4D flow CMR peak velocity in discordant TTE assessment of AS. Third, this study sought to test the hypothesis that 4D flow CMR peak velocity was more predictive of subsequent valve intervention than equivalent TTE indices.

## Methods

### Study cohort

The PREFER-CMR registry was used to identify suitable participants for inclusion in this study (NCT05114785, first posted 10 November 2021).^12^ Participants who consecutively had CWD TTE followed by 4D flow CMR between February 2021 and January 2022 were included.

### Inclusion criteria

The study required participants to be at least 18 years old and to have received a diagnosis of AS via TTE. Patients with contraindications to CMR, such as those with implanted defibrillators incompatible with CMR, claustrophobia and end-stage renal failure (estimated glomerular filtration rate (eGFR)<30 mL/min/1.73 m^2^), were excluded. Patients who had undergone prior AV interventions, either catheter-based or surgical, were also excluded from the study.

Although atrial fibrillation (AF) is known to affect the accuracy of 4D flow CMR measurements due to beat-to-beat variability, patients with AF were not excluded from participation. This decision reflected the pragmatic approach to study design and recognition that patients with AS frequently have concurrent arrhythmias. As such, including patients with AF ensured that study findings were more generalisable to a real-world patient population.

A pragmatic opt-out informed consent was obtained from all patients included in the study.^13^ This study was conducted in compliance with the principles contained within the Declaration of Helsinki.^14^

### Echocardiography

The assessment of AS using TTE followed the guidelines published by the British Society of Echocardiography (BSE) and was reported in adherence to the BSE guideline protocol.^2 15^ The severity of AS was graded based on V_Peak_ and mean AV velocity in accordance with the guidelines established by the European Society of Cardiology.^3^

V_Peak_ (m/s) was measured using CWD TTE, with the highest velocity recorded from any acoustic window used for analysis. In patients with AF, to address beat-by-beat variability, we took multiple consecutive CWD measurements over a series of at least five cardiac cycles. We typically choose cycles with relatively consistent R–R intervals. AV mean velocity was calculated using the spectral Doppler signal to trace the velocity-time integral of the aortic flow profile. The mean AV velocity was then calculated by averaging the instantaneous peak velocities occurring during systole.

### Cardiovascular magnetic resonance protocol

CMR studies were performed on a 1.5 Tesla Magnetom Sola (Siemens Healthineers, Erlangen, Germany) system equipped with BioMatrix Body 18 coil technology. The CMR protocol comprised baseline surveys, cine imaging (including vertical long-axis, horizontal long-axis, short-axis contiguous left-ventricular volume stack, three-chamber and aortic root), native T1-mapping and 4D flow acquisition. The calculation of volumetric indices and T1 mapping was performed according to established protocols and guidelines, which are described in detail in online supplemental materials.

Cine images were acquired during end-expiratory breath-hold with a balanced steady-state free precession, single-slice breath-hold sequence. 30-phase cine images had a contiguous slice thickness of 8 mm for the short-axis stack. Cine imaging parameters were echo time (TE) 1.13 ms, repetition time (TR) 2.71 ms, flip angle (80°), field of view (FOV) 360×289 mm^2^ and GeneRalized Autocalibrating Partial Parallel Acquisition (GRAPPA) factor 2.

### Four-dimensional flow CMR acquisition

For 4D flow, 30 phases throughout the cardiac cycle were acquired to ensure consistency with the cines. The temporal resolution was 40 ms, TR 4.98 ms, TE 1.13 ms, FOV 200×256 mm^2^, flip angle (5°), GRAPPA acceleration in the phase-encoding direction with a factor of 2 and slide direction of 1. The ECG was retrospectively gated with free breathing to avoid diastolic temporal blurring. A three-dimensional volume with complete coverage of the thoracic aorta was acquired in the axial plane.

### Four-dimensional flow CMR velocity assessment

CMR images were postprocessed and analysed using CVI42, V.5.14 (Circle Cardiovascular Imaging, Calgary, Canada). The 4D flow analysis pipeline was conducted independently and blinded to the echocardiographic CWD assessment, and conversely, the CWD analysis was performed without knowledge of the 4D flow results.

All three phase directions were screened for aliasing artefacts and, if present, manually corrected using established phase unwrapping methods.^16^ To determine V_Peak_, an analysis plane was set perpendicular to the forward flow jet at the level and phase recording the highest flow velocity values. The grading of AS severity using 4D flow CMR was based on peak aortic velocity measurements, similar to TTE. However, unlike TTE, which relies on CWD assessment and requires optimal alignment with the flow jet, 4D flow CMR provides a three-dimensional assessment of peak velocity independent of angle alignment, potentially offering a more accurate representation of transvalvular velocity.

**Figure 1:**
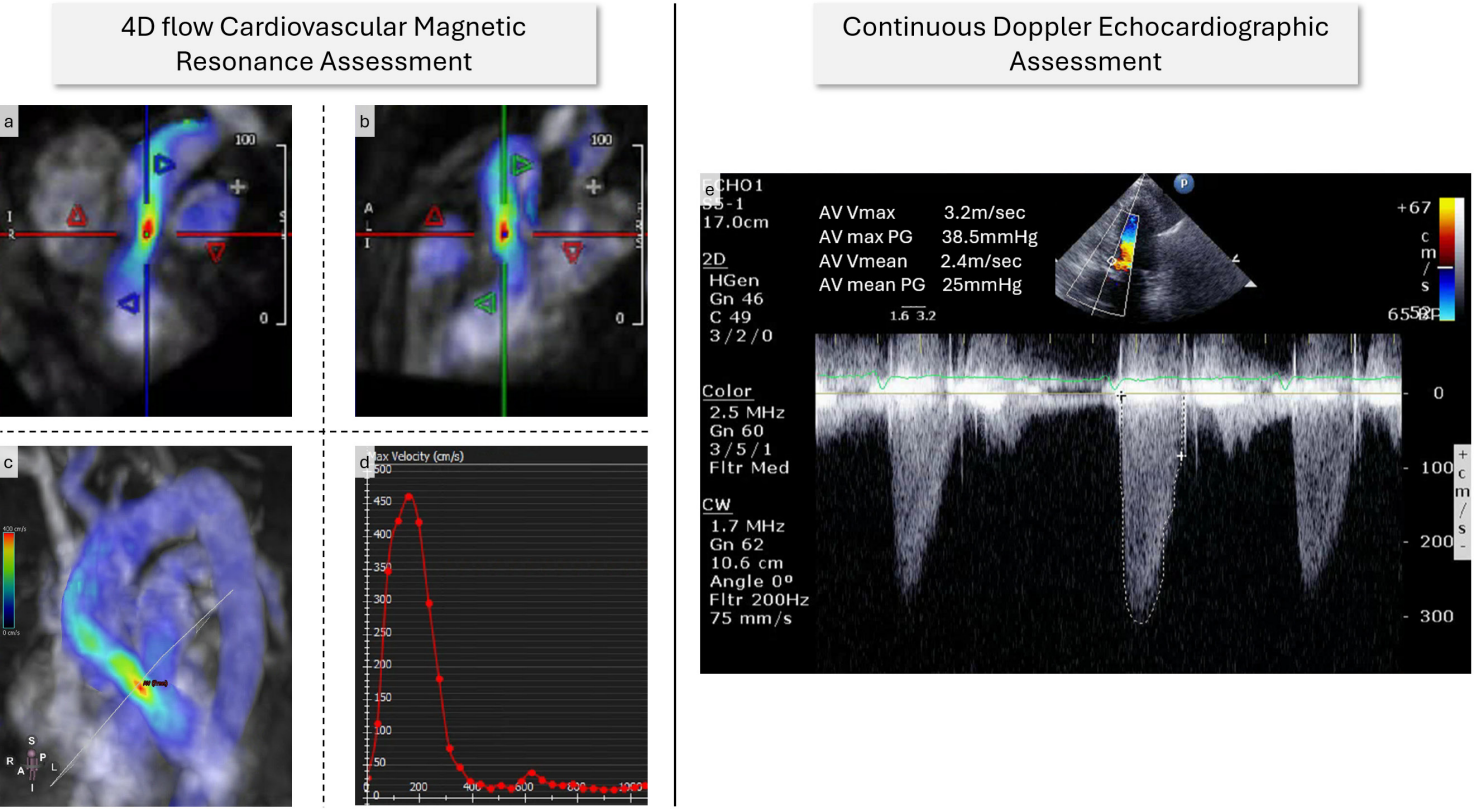
A case example from this study. (**a–b**) Demonstrates V_Peak_ assessment using 4D flow data superimposed on two orthogonal views of the aortic valve. (**c**) Three-dimensional visualisation of the plane where peak aortic valve velocity above 4 m/s can be identified. (**d**) 4D flow AV V_Peak_ was 4.7 m/s in this case. (**e**) CWD TTE assessment of the aortic valve stenosis with a V_Peak_ of 3.2 m/s. AV, aortic valve; CWD, continuous wave Doppler; TTE, transthoracic echocardiography; V_Peak_, peak aortic valve velocity.

An example imaging workflow through TTE and 4D flow CMR is presented in figure 1.

### Outcomes

This study focused on evaluating the agreement of 4D flow CMR compared with CWD TTE in patients with AS and assessing the potential of 4D flow CMR to enhance the accuracy of AS diagnosis and guide clinical decision-making.

### Statistical analysis

Data analyses were conducted using MedCalc, V.20.011 (MedCalc Software, Ostend, Belgium). Normality was investigated using the Shapiro-Wilk test for key variables—CWD and 4D flow derived V_Peak_. Continuous variables are presented as median±IQR or mean±SD. The independent t-test was used to compare continuous variables between patients managed conservatively and those treated with valve intervention, while the χ² test was employed for categorical variables. To compare V_Peak_ measurements between 4D flow CMR and TTE, Bland-Altman plots were used, and statistical significance was determined using the paired samples t-test. Correlation analysis of V_Peak_ values between the two modalities was performed using Pearson correlation. The level of agreement between 4D flow CMR and TTE AS severity grading was calculated using χ² statistics. Receiver operator characteristic (ROC) analysis was performed to determine the peak aortic velocity threshold significantly associated with AV intervention, with the model performance reported as the area under the ROC curve (AUC). Kaplan-Meier plots were used to demonstrate the aortic intervention-free intervals using the thresholds previously determined from the ROC analysis. Cox-regression survival analysis was used to evaluate the incremental value of 4D flow V_Peak_. The statistical analyses were performed with a significance level of p<0.05.

### Sample size calculations

To compare the predictive power of 4D flow CMR and TTE for AV intervention in AS, the sample size was calculated using a prior study’s effect size: a correlation of −0.45 between 4D flow CMR peak pressure gradient and 6-min walk test.^9^ Assuming a method correlation of 0.8, a sample of about 29 achieves 80% power to detect a correlation difference of 0.35–0.45 at a 0.05 significance level. This reflects functional capacity’s role in intervention timing, adjusted for real-world variability.

## Results

### Study population

30 participants with AS underwent both TTE and 4D flow CMR as part of this study. Of the 30 participants, 17 (57%) underwent AV intervention, while 13 (43%) were managed conservatively during a mean follow-up period of 8 months. One participant had congenital bicuspid AV (BAV). There was a 3-month median interval between CMR and echocardiography, from which 27% of cases had CMR first and then echocardiography.

The baseline characteristics of the participants are summarised in table 1. In this cohort, patients referred for AV intervention (n=17) were significantly younger than those not requiring intervention (72.1±6.8 vs 79.9±5.1 years, p=0.0017). No significant differences were observed in height, weight, gender distribution or comorbid conditions such as hypertension, diabetes mellitus, AF, ischaemic heart disease, myocardial infarction, chronic obstructive pulmonary disease and smoking status. Laboratory values also showed no significant differences except for the eGFR, which was higher in the intervention group (72.2±18.6 vs 56.3±16.6 mL/min/1.73 m², p=0.0219). Other biomarkers, including haemoglobin, N-terminal pro–B-type Natriuretic Peptide (NT-proBNP), creatinine and urea, did not differ significantly between groups.

**Table 1:** Study demographics and results of baseline investigations for patients

Variable	No AV intervention recommended	Referred for AV intervention	P value
N	13	17	
Age (years)	79.9±5.1	72.1±6.8	0.0017*
Height (cm)	170.9±12.9	173.2±10.0	0.5943*
Weight (kg)	73.9±20.9	89.7±22.8	0.0615*
Male (N (%))	9 (69)	11 (65)	0.7978†
NYHA class I	2 (15%)	5 (29%)	0.3232†
NYHA class II	7 (54%)	5 (29%)	
NYHA class III	2 (15%)	6 (35%)	
NYHA class IV	2 (15%)	1 (6%)	
CCS class I	5 (38%)	10 (59%)	0.8849†
CCS class II	3 (23%)	3 (18%)	
CCS class III	1 (8%)	1 (6%)	
CCS class IV	1 (8%)	2 (12%)	
Hypertension N (%)	9 (69)	11 (65)	0.7978†
Diabetes mellitus N (%)	11 (85)	11 (65)	0.2296†
Atrial fibrillation N (%)	8 (62)	10 (59)	0.8824†
Ischaemic heart disease N (%)	5 (38)	7 (41)	0.8824†
Myocardial infarction N (%)	10 (77)	15 (88)	0.4179†
Chronic obstructive pulmonary disease N (%)	11 (85)	14 (82)	0.8713†
Smoking N (%)	10 (77)	12 (71)	0.7023†
Haemoglobin (g/L)	154.5±76.8	133.5±17.9	0.2831*
NT-proBNP (pg/mL)	6810.4±10 299.1	1639.2±3731.1	0.1052*
Creatinine (µmol/L)	107.8±36.3	85.8±24.0	0.0560*
Urea (mmol/L)	9.3±5.9	6.9±4.4	0.2260*
eGFR (mL/min/1.73 m²)	56.3±16.6	72.2±18.6	0.0219*

Data are: mean±SD deviation.

* T-test.

†Χ ² test.

AV, aortic valve; CCS, Canadian Cardiovascular Society classification of angina pectoris; eGFR, estimated glomerular filtration rate; NT-proBNP, N-terminal pro–B-type Natriuretic Peptide; NYHA, New York Heart Association Functional Classification.

In the CMR assessment, patients referred for AV intervention demonstrated significantly higher left ventricular ejection fraction (p=0.02) and right ventricular ejection fraction (p=0.004) compared with those not requiring intervention. Additionally, 4D flow V_Peak_ was significantly higher in the intervention group (p<0.001) (table 2).

**Table 2:** CMR and echocardiographic assessment in the whole study population

Variable	No AV Intervention Needed	Referred for AV intervention	P value*
CMR assessment			
Left ventricular end-diastolic volume (mL)	203.3±92.5	171.8±71.7	0.3010
Left ventricular end-systolic volume (mL)	116.4±97.3	74.7±66.8	0.1738
Left ventricular stroke volume (mL)	86.8±36.0	102.5±34.5	0.2360
Left ventricular mass (g)	167.0±47.1	174.8±59.6	0.7007
Left ventricular ejection fraction (%)	47.4±19.4	62.5±12.8	0.0157
Right ventricular end-diastolic volume (mL)	184.4±88.8	172.5±71.7	0.6877
Right ventricular end-systolic volume (mL)	102.8±64.8	78.5±47.0	0.2435
Right ventricular stroke volume (mL)	81.3±34.2	93.9±30.9	0.2994
Right ventricular ejection fraction (%)	45.2±8.9	55.9±9.7	0.0042
Native T1 (ms)	1052.0±69.1	1043.5±73.9	0.7537
4D flow V_Peak_ (m/s)	2.7±0.7	4.2±0.7	<0.0001
Transthoracic echocardiographic assessment			
Interventricular septal diameter (mm)	13.2±3.8	12.8±2.0	0.7192
Left ventricular internal diameter in diastole (mm)	49.5±9.1	48.0±8.9	0.6469
Left ventricular posterior wall thickness in diastole (mm)	11.9±3.3	11.6±2.3	0.7897
Early diastolic velocity (m/s)	1.0±0.3	1.2±0.5	0.3126
Atrial systolic velocity (m/s)	0.8±0.3	1.3±0.7	0.1286
Medial e' velocity (cm/s)	6.1±3.1	6.2±2.1	0.9324
Lateral e' velocity (cm/s)	9.4±2.7	9.5±3.9	0.9782
Tricuspid annular plane systolic excursion (mm)	5.4±7.8	6.7±9.3	0.7218
Tricuspid regurgitation peak (m/s)	1.9±1.7	2.2±1.6	0.7158
CWD V_Peak_ (m/s)	2.6±0.5	3.4±0.7	0.0025
CWD mean velocity (m/s)	2.1±0.5	2.7±0.7	0.0077
Dimensionless Velocity Index (DVI)	3.9±11.3	0.6±1.0	0.2545

* T-test.

CMR, cardiovascular MRI; CWD, continuous wave Doppler; V_Peak_, peak aortic valve velocity.

In the transthoracic echocardiographic assessment, patients referred for intervention had significantly higher CWD V_Peak_ (p=0.003) and mean velocity (p=0.008). Other parameters, including ventricular volumes, mass, septal thickness and other Doppler velocities, did not differ significantly between groups.

### Correlation between 4D flow CMR and TTE V_Peak_ measurement

**Figure 2:**
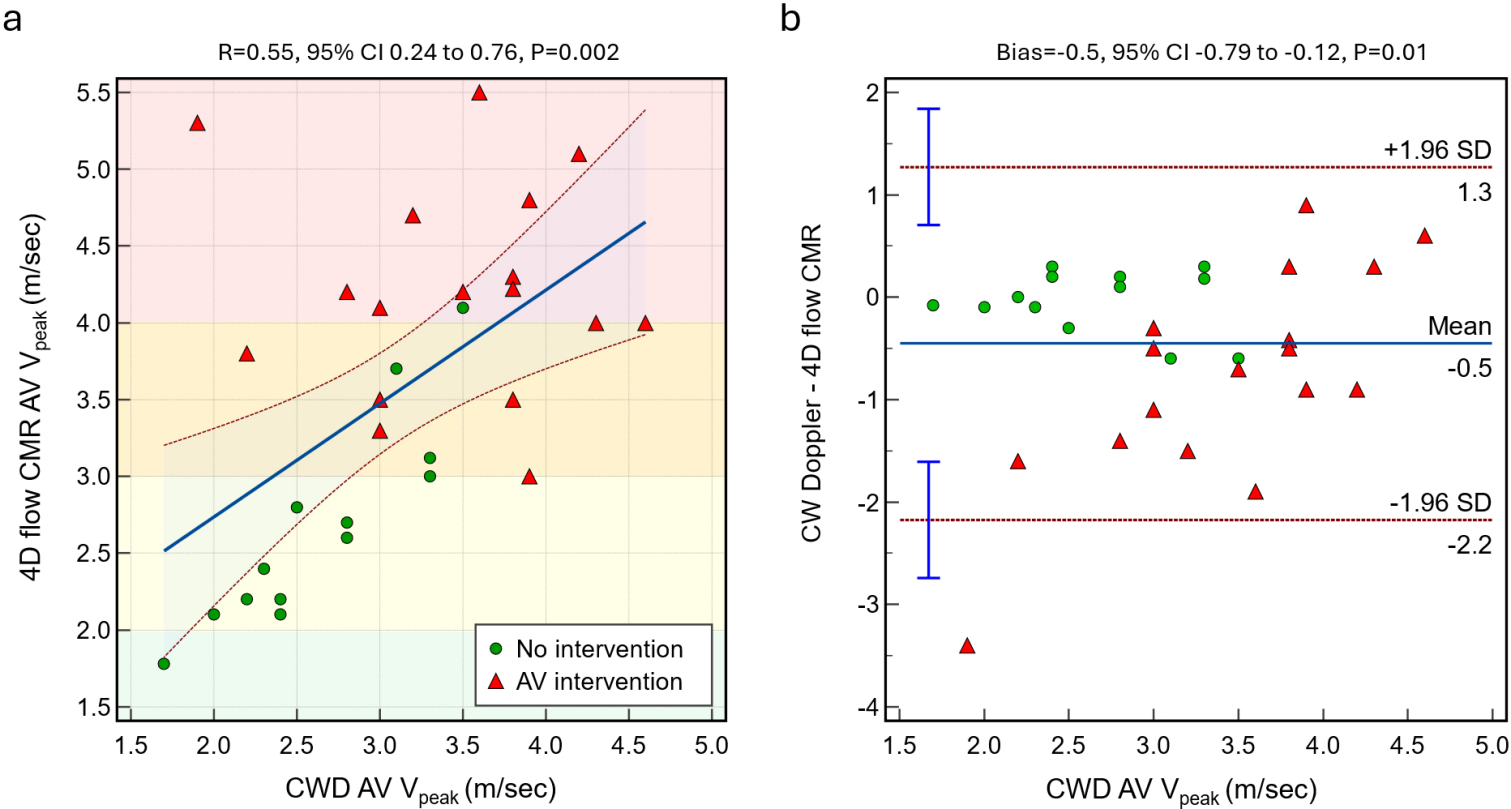
(**a**) Scatterplot between CWD and 4D flow CMR AV V_Peak_. (**b**) Bland-Altman plot shows a significant bias between methods. Green circles (no intervention cohort) and red triangles (intervention cohort). AV, aortic valve; CMR, cardiovascular MRI; CWD, continuous wave Doppler; V_Peak_, peak aortic valve velocity.

A moderate positive correlation was observed between CWD V_Peak_ and 4D flow CMR V_Peak_ (R=0.55, 95% CI: 0.24 to 0.76, p=0.002), with distinct distributions for patients with and without AV intervention (figure 2). The agreement analysis showed a mean bias of −0.5 m/s (95% CI: −0.79 to −0.12, p=0.01), suggesting that CWD slightly underestimated the V_Peak_ compared with 4D flow CMR. The limits of agreement were 1.3 m/s and −2.2 m/s, indicating variability between methods, particularly in the intervention group.

### Predictors of aortic valve intervention

During the ROC analysis (figure 4), 4D flow CMR V_Peak_ demonstrated significant predictive value for a longer AV intervention-free period (AUC=0.78, 95% CI: 0.57 to 0.92, p=0.01). A V_Peak_ threshold of 3.5 m/s had a sensitivity and specificity of 80% in identifying patients who may benefit from early valvular intervention. In contrast, TTE V_Peak_ assessment did not show a significant association with follow-up AV intervention (AUC=0.71, 95% CI 0.49 to 0.87, p=0.08).

### Receiver operating characteristic analysis

**Figure 3:**
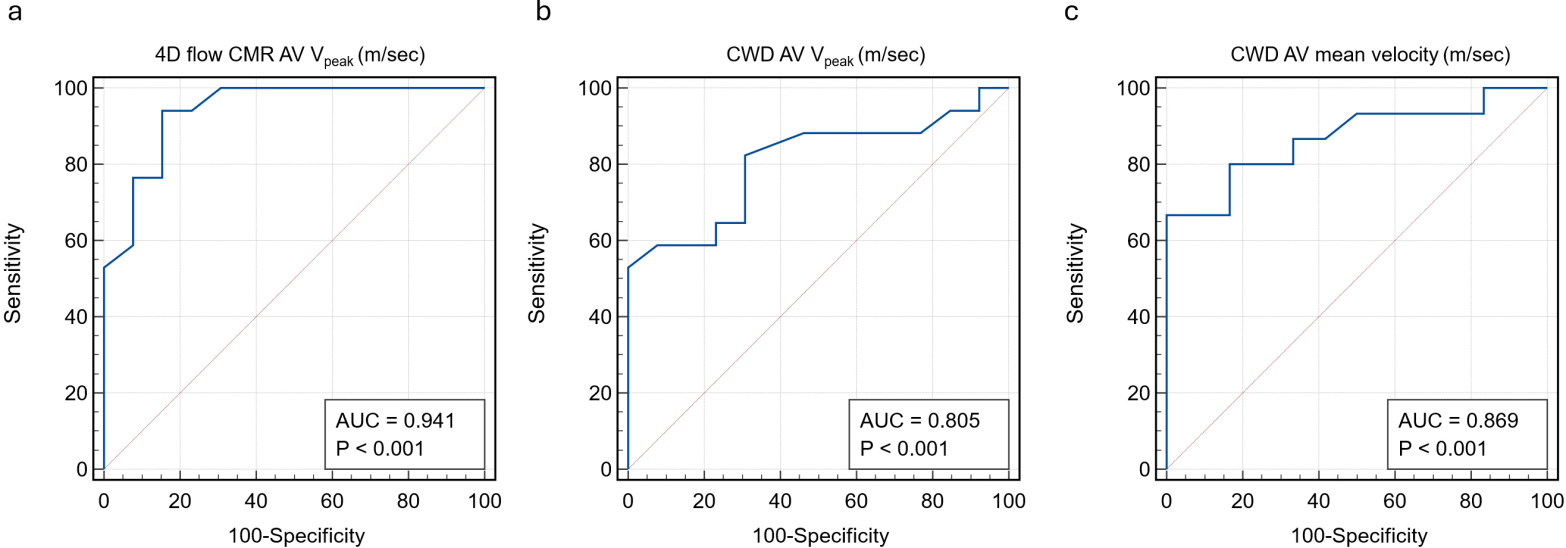
Receiver operating characteristic curves for predicting AV intervention based on 4D flow AV V_Peak_ (**a**), CWD V_Peak_ (**b**) and CWD mean AV velocity (**a**). AUC, area under the curve; AV, aortic valve; CMR, cardiovascular MRI; CWD, continuous wave Doppler; V_Peak_, peak aortic valve velocity.

ROC analysis for predicting AV intervention demonstrated that 4D flow AV V_Peak_ yielded an AUC of 0.941 (p<0.001) (figure 3). CWD V_Peak_ showed a lower, yet strong predictive value, with an AUC of 0.805 (p<0.001). Similarly, CWD AV mean velocity also demonstrated high predictive accuracy with an AUC of 0.869 (p<0.001).

### Kaplan-Meier curve analysis

**Figure 4:**
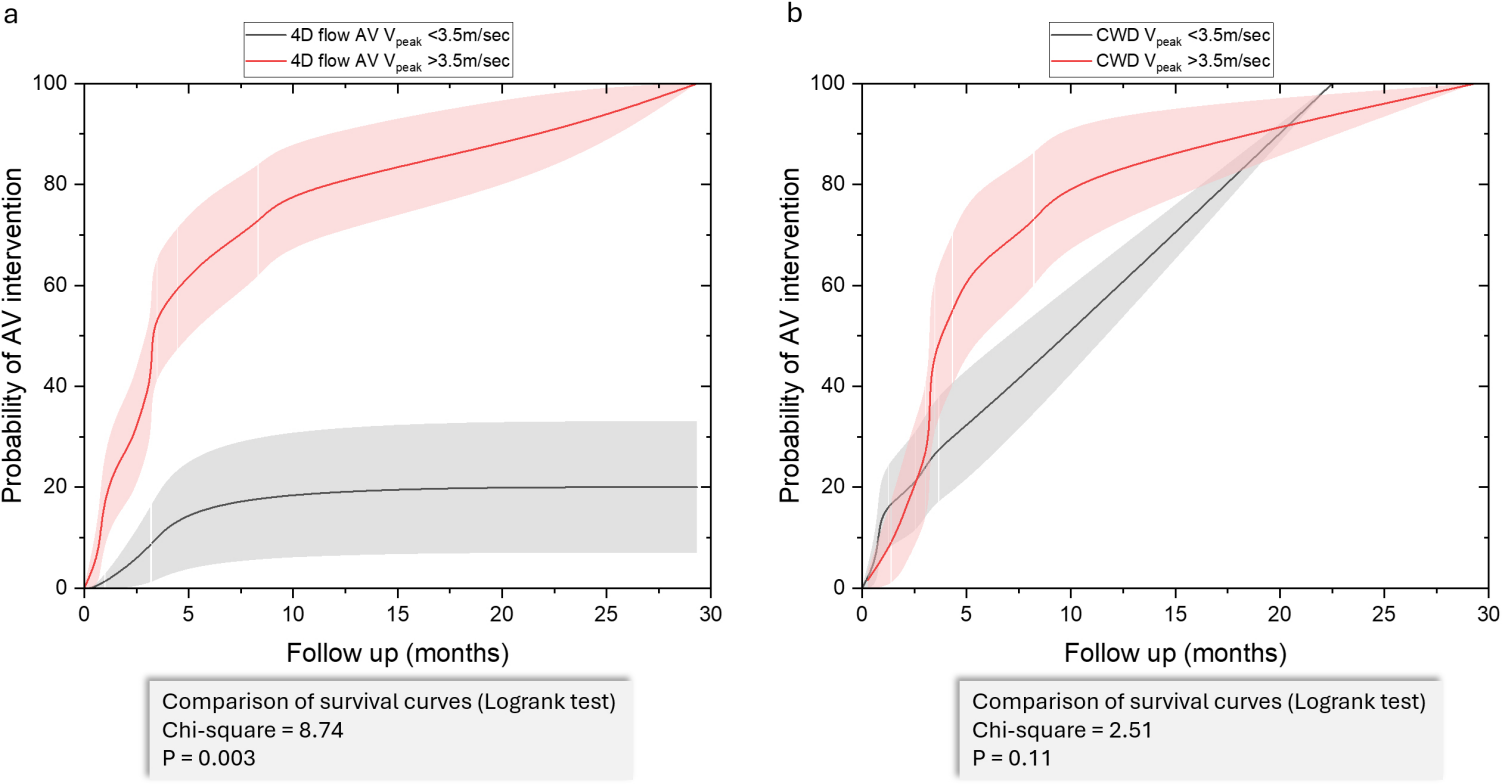
Kaplan-Meier curves for AV intervention probability based on 4D flow AV V_Peak_ (**a**) and CWD V_Peak_ (**b**). A 4D flow AV V _Peak_>3.5 m/s was significantly associated with higher intervention probability (p=0.003), while CW Doppler peak velocity >3.5 m/s showed no significant difference (p=0.11). B-spline smoothing was applied to the curves to enhance visualisation. AV, aortic valve; CWD, continuous wave Doppler; V_Peak_, peak aortic valve velocity.

Kaplan-Meier analysis comparing the probability of AV intervention based on 4D flow AV V_Peak_ and CWD V_Peak_ showed significant differences in intervention likelihood over time (figure 4). Patients with a 4D flow AV peak velocity greater than 3.5 m/s had a markedly higher probability of requiring AV intervention than those with a peak velocity below this threshold (Log-rank test: χ²=8.74, p=0.003). In contrast, for CWD V_Peak_, this difference did not reach statistical significance (Log-rank test: χ²=2.51, p=0.11).

### Cox-regression survival analysis

**Figure 5:**
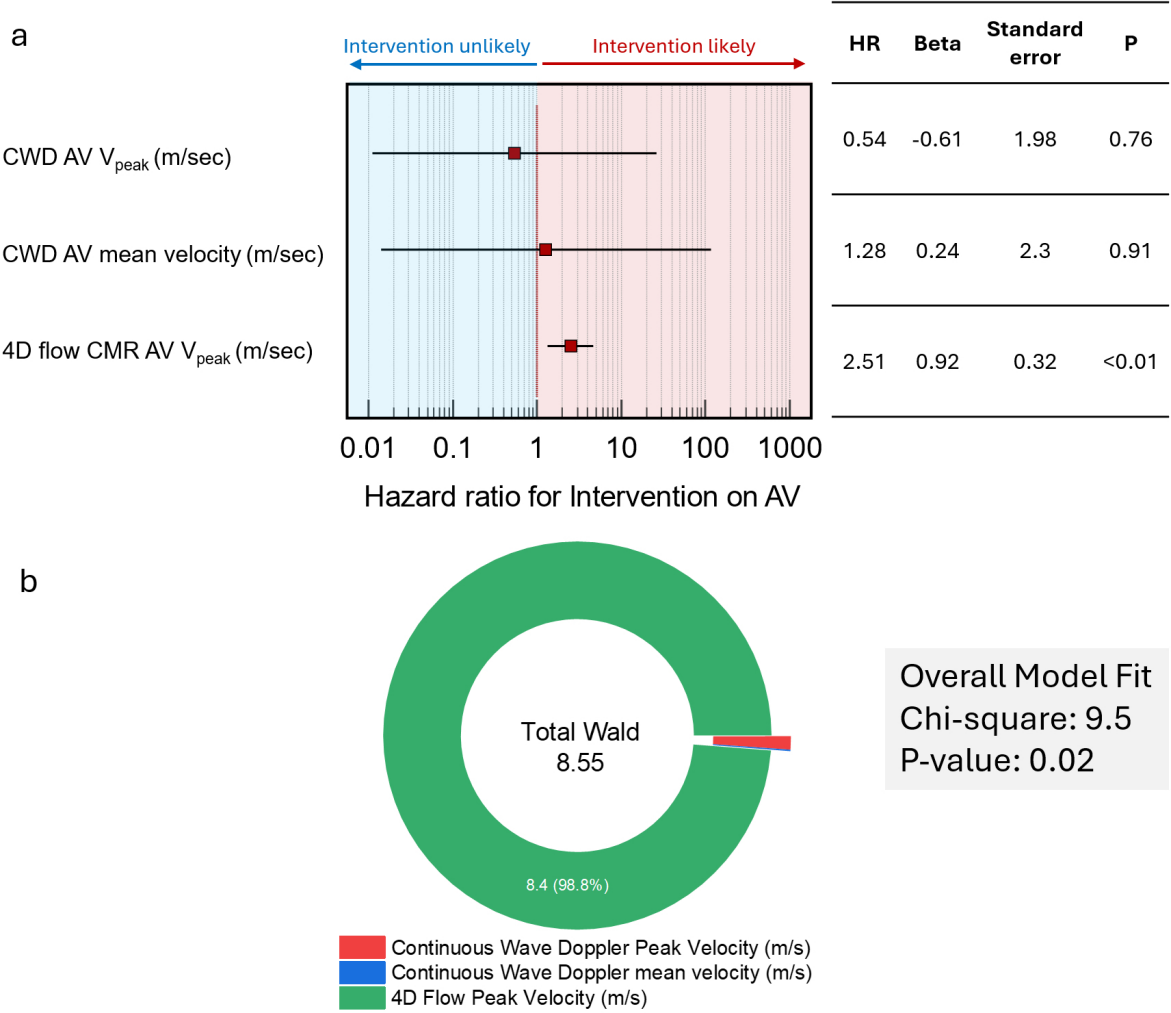
Cox regression analysis for AV intervention. (**a**) Shows forest plot, with 4D flow V_Peak_ significantly associated with intervention (HR=2.51, p<0.01), while CWD peak and mean velocities are not significant predictors. (**b**) Displays the total Wald statistic, with 4D flow V_Peak_ contributing 98.8% of the model’s explanatory power (total Wald=8.55). AV, aortic valve; CMR, cardiovascular MRI; CWD, continuous wave Doppler; V_Peak_, peak aortic valve velocity.

Multivariate Cox regression survival analysis identified 4D flow AV V_Peak_ as a significant predictor of AV intervention, with a HR of 2.51 (p<0.01), indicating a strong association with intervention likelihood (figure 5). In contrast, CWD V_Peak_ and mean velocity did not demonstrate significant predictive value, with HRs of 0.54 (p=0.76) and 1.28 (p=0.91), respectively. The overall model fit was statistically significant, with a χ² value of 9.5 and p=0.02. The Wald statistical analysis showed that 4D flow peak velocity accounted for most of the model’s explanatory power, contributing 98.8% of the total Wald value (8.4 out of 8.55), further emphasising its predictive strength.

## Discussion

### Main findings

This study provides unique insights into the complementary value of 4D flow CMR in patients with a common yet diagnostically challenging clinical entity. The main findings of the study include that 4D flow CMR AV V_Peak_ not only correlates with echocardiographic CWD V_Peak_ assessment, but is likely to be superior due to better spatial recognition of the true peak transvalvular velocity. Moreover, this study suggests that this 4D flow CMR V_Peak_ can predict AV intervention better than CWD, which could potentially improve diagnosis, monitoring and the optimal timing of AV intervention.

In the current study, we found a modest correlation between TTE and 4D flow CMR V_Peak_, similar to previous studies conducted by our group.^9^ Even though it is a modest correlation, in the present study, we noted that CWD underestimated the V_Peak_, which is in contrast to our previous work, where we observed no statistical difference. We postulate this is due to multiple factors. First, in the present study, we are using different MRI hardware (Siemens vs Philips). Second, in the present study, which is more representative of real-world imaging, there is a possibility that CWD was underestimated due to the Doppler alignment issue to the aortic flow jet—especially in very eccentric jets. Hence, 4D flow might play a complementary role, especially in those patients where CWD alignment is an issue and is likely to circumvent the need to progress to semi-invasive transoesophageal echocardiography.

### Benefits of CMR in the assessment of aortic stenosis

It is crucial to consider that the benefits of CMR extend beyond the isolated calculation of pressure gradients and velocities. CMR offers a gold standard assessment of ventricular function and the ability to visualise and quantify concurrent valvular lesions, particularly those with multiple or eccentric jets.^17^ Additionally, the ability to retrospectively and flexibly quantify and visualise flow without being restricted to two-dimensional planes is a significant advantage of 4D CMR. However, these benefits must be weighed against the limitations of 4D flow CMR, which include limited spatiotemporal resolution, comparatively longer image acquisition times and the requirement for specialised and time-consuming postprocessing.^18^

The correlation between transcatheter invasive and 4D flow CMR assessment of pressure gradients has been shown to be good, with one study demonstrating the prognostic advantage of 4D flow-derived gradient versus TTE, as evidenced by LV remodelling post-AV surgical intervention.^9^ In addition to the ability of CMR to precisely assess LV function and detect the presence of fibrosis, a useful biomarker of LV decompensation even in the absence of symptoms,^19^ 4D flow CMR can evaluate the haemodynamic consequence of pathological blood flow through the valve into the ascending aorta through the quantification of kinetic energy, flow displacement, wall shear stress and turbulent kinetic energy.^20 21^ Comprehensive assessment of such advanced markers may be helpful in patients with paradoxical low-flow low-gradient AS, a frequent diagnostic challenge in clinical practice and an area of research with notable sparsity of research data.^22^ These advanced markers can help differentiate between true and pseudo-severe AS and provide information on the mechanisms driving reduced transvalvular flow and prognostically significant LV dysfunction.^23^ Although not highly prevalent in our study cohort, patients with a BAV present additional diagnostic challenges, for which 4D flow CMR can interrogate abnormal flow patterns and imaging markers prognostic for subsequent aortic dilation.^24^ In this study, an ROC-derived threshold of 3.5 m/s provided the optimal balance of sensitivity and specificity for predicting AV intervention, potentially reflecting a trend towards earlier intervention in patients with symptoms or those at risk of rapid disease progression.

Although some exploratory works have been published on the concordance between TTE and 4D flow CMR indices across the spectrum of AS severity,^9 25^ no study has been conducted on the complementary value of 4D flow CMR in discordant TTE assessment. Further exploration of the role of both phase-contrast and 4D flow CMR in discordant TTE assessment, with a focus on the incremental value in the classification of AS severity, would be valuable. Additionally, there is a clinical need to determine the survival benefit of AVR based on decisions made from 4D flow CMR analysis in prospectively designed clinical trials.

While our previous research has demonstrated the reliability and accuracy of flow volume measurement in AF,^26^ peak velocity assessment by 4D flow in AF underestimated the values due to the susceptibility of 4D flow to R-to-R variability and averaging of peak velocity over several heartbeats. It is important to note that even TTE assessment can prove to be challenging in patients with arrhythmias due to the inconsistency of peak velocity measurements.^27^

Although 4D flow is unlikely to replace TTE CWD in the comprehensive assessment of AS, it can provide additional information that complements the advantages of TTE. The effect of AV disease on haemodynamics is complex and poorly understood. While new 4D flow metrics have the potential to improve the characterisation of aortic disease, our study shows that simple flow metrics generated from 4D flow CMR have clinical value in a real-world clinical setting. Further research is needed to evaluate the role of both simple flow and advanced haemodynamic metrics, with a specific focus on risk stratification in discrepant cases and personalised therapeutic decision-making for optimal patient outcomes.

### Limitations

The insights gained from this study should be interpreted in light of the limitations associated with the study methodologies. First, the study was exploratory in nature and conducted at a single centre. Second, the follow-up period of 8 months (mean) was relatively short and did not allow for the evaluation of postintervention outcomes such as heart failure hospitalisation or mortality. Third, although operator variability in echocardiographic assessment might introduce bias, it accurately mirrors real-world practice since most specialist centres employ a team of sonographers with diverse skill levels. Fourth, the decision to proceed with AV intervention or conservative management was ultimately the prerogative of the multidisciplinary team, for which imaging results were considered alongside a catalogue of patient and service factors. However, this reflects real-world clinical practice and reinforces that patients are complex, comorbid and require individualised approaches to care.

Similarly, the interval between TTE and 4D-flow CMR introduces the potential for natural disease progression between scans, thereby limiting the ability to draw definitive conclusions regarding the agreement of peak velocity assessment between modalities. However, clinically, patients remained in similar symptom burden, confirming the unlikely significant change. Future research should address these questions to improve the clinical utility of these imaging modalities.

## Conclusion

4D flow CMR-derived V_Peak_ assessment is superior to echocardiographic CWD assessment for predicting timing of AV intervention.

## Supplementary material

10.1136/openhrt-2024-003081 online supplemental file 1

## Data Availability

Data are available upon reasonable request.
