# Supplementary material for: Four-dimensional flow provides incremental diagnostic value over echocardiography in aortic stenosis
Source: Open Heart. 2025 May 7;12(1):e003081. doi: 10.1136/openhrt-2024-003081 (PMC12060880; doi:10.1136/openhrt-2024-003081)
Supplement: online supplemental file 1 [file openhrt-12-1-s001.docx]

**Supplementary methods**

**Volumetric assessment**

For the volumetric assessment of the left and right ventricle, endocardial and epicardial borders were drawn in short-axis cines at end-diastole and end-systole using the automated software tool and were manually corrected as required. The papillary muscles were manually excluded. LV and right ventricular (RV) end-diastolic volume, end-systolic volume, stroke volume, ejection fraction, and LV mass were computed. Left atrial (LA) contours were constructed using the automated tool, and manually corrected. LA volume was determined in the LV end-systolic phases in two-chamber and four-chamber cine images and was recorded as a mean value.

**T1 mapping**

T1-mapping was performed in the mid-ventricular slice using the Modified Look-Locker Inversion Recovery (MOLLI) sequence. The acquisition parameters used were as follows:

| **Parameter** | **Value** |
| --- | --- |
| Slice thickness (mm) | 8 |
| Flip angle (°) | 35 |
| TE | 1.01 |
| TR | 2.42 |
| GRAPPA factor | 2 |

**Abbreviations**: FOV – field of view; TE – echo time; TR – repetition time

For T1 mapping, regions of interest (ROI) were manually contoured in the left ventricular cavity and myocardium for native images.
